# Observations of the warm-tongue circulation in the northern East China Sea

**DOI:** 10.1038/s41598-019-57148-6

**Published:** 2020-01-14

**Authors:** Hojun Lee, Kyungjae Lee, SungHyun Nam, Jae-Hak Lee

**Affiliations:** 1 School of Earth and Environmental Sciences, Seoul National University, Seoul, Republic of Korea; 2 Research Institute of Oceanography, Seoul National University, Seoul, Republic of Korea; 3 Korea Institute of Ocean Science and Technology, Busan, Republic of Korea

**Keywords:** Physical oceanography, Physical oceanography

## Abstract

A subsurface thermohaline front semi-permanently formed in association with near-bottom cyclonic circulation in the northern East China Sea was newly found from detailed hydrographic data collected during two cruises in February 2017 (winter) and April 2018 (spring) along with supplementary satellite remote sensing and historical hydrographic data. An alternate intruding frontal structure in water properties was observed across the cyclonic circulation in both seasons as formed by two contrasting water masses—low-temperature and low-salinity (i.e., low spiciness) water transported by the East China Sea Current and high-temperature and high-salinity (i.e., high spiciness) water transported by the Tsushima Warm Current. Consistent structures were confirmed from current observations during the two cruises, historical hydrographic observations, and satellite altimetry-derived sea surface height and surface frontal structure, indicative of retroflection of the Cheju Warm Current as deemed by the seasonal development of thermal stratification in spring. Our results reveal significant heat and material exchanges between the open Pacific and the broad shelf, particularly via diapycnal mixing and cross-front transports associated with across-front flow and cyclonic circulation, in the northern East China Sea.

## Introduction

Understanding the structure of the thermohaline front and associated circulation in the northern East China Sea is of great importance because the northern East China Sea is located in the middle of heat and material exchange between the open Pacific and the neighbouring marginal seas such as the Yellow Sea, East China Sea, and coastal waters around Korea^1–3^. The formation and dissipation of the thermohaline front in the northern East China Sea are very closely linked with the variation of the seasonal currents^2^. The currents and circulation of the northern East China Sea are driven and modified by four factors: the Kuroshio main stream, northeast Asian Monsoon, tides, and the buoyancy formed by surface solar heating and freshwater discharge^4^. Among these factors, the northeast Asian Monsoon has the prominent effect on the currents and circulation of the northern East China Sea^4,5^. The northeast Asian Monsoon is dominated by northerly winds in winter and southerly winds in summer^1^.

**Figure 1:**
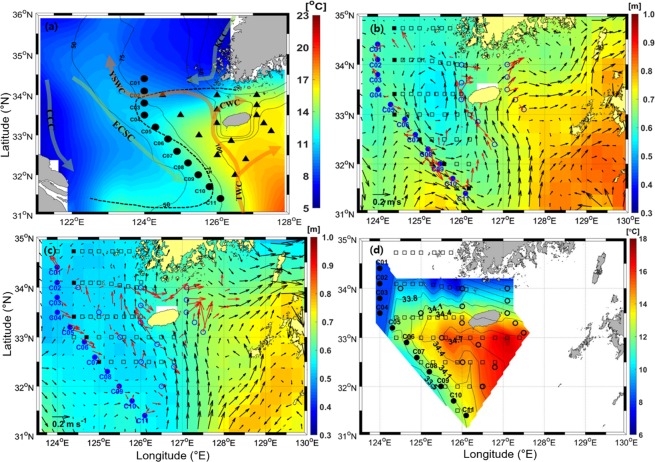
(**a**) Schematics of regional circulation with bathymetry (contours), SST (colours), and hydrographic observation stations (closed circles and triangles); C-line stations are indicated by filled circles with labels. The SST averaged from January to March 2017 is indicated by the colour bar on the right. Horizontal currents (red arrows) observed at mid-depth (40 m) after removal of tidal currents during (**b**) Feb. 6–14, 2017 and (**c**) Apr. 20–30, 2018, where satellite altimetry-derived surface geostrophic currents averaged over corresponding periods (black arrows) are superimposed with sea surface height (colours). **(d**) Horizontal distribution of climatological mean of conservative temperature (Θ, colours) and absolute salinity (S_A_, contours) at the mid-depth (40 m) for February. In (**a**), the S-shaped Cheju-Yangtze Front (CYF) and regional currents (ECSC: East China Sea Current, CWC: Cheju Warm Current, KCC: Korean Coastal Current, TWC: Tsushima Warm Current, and YSWC: Yellow Sea Warm Current) are indicated by dashed lines and red-coloured (warm and saline) and green-coloured (cold and fresh) arrows. Figures were generated by H.J. Lee using MATLAB R2017b (http://www.mathworks.com).

During the winter monsoon, the Korea Coastal Current (KCC) and the China Coastal Current (CCC) transport relatively low-temperature and low-salinity waters southward along the west coast of Korea and the east coast of China, respectively^4^. In contrast, the Yellow Sea Warm Current (YSWC) intermittently transports high-temperature and high-salinity water of Tsushima Warm Water (TWW) origin northward toward the west trough of the Yellow Sea when the northerly wind bursts^6,7^. The YSWC branches from the Tsushima Warm Current (TWC) to the west of Cheju Island. The divergence formed by the southward wind-driven CCC and the northward YSWC drives the CCC to move in the southeast direction, creating an offshore East China Sea Current (ECSC)^8^. The ECSC, as a cross-shelf current, carries a considerable amount of coastal fine-grain sediments from the entrance of the old Huanghe (Yellow River) mouth off toward Cheju Island, thereby contributing to local material cycles^8,9^. The Cheju Warm Current (CWC) originating from the TWC flows through the Cheju Strait clockwise throughout the year (Fig. 1a)^4,10^. From winter to summer monsoons, the YSWC that enters the interior of the Yellow Sea disappears, and a large amount of low-salinity water from the Changjiang Diluted Water flows northeastward toward Cheju Island in summer^4,11^. During the summer monsoon, the CCC and KCC flow northward along the Chinese and Korean coasts, respectively, owing to southerly winds^4,12^. During winter, the ECSC and KCC transporting low-temperature and low-salinity water and the CWC and YSWC transporting high-temperature and high-salinity water converge in the northern East China Sea to form a strong thermohaline front.

This thermohaline front is called the Cheju-Yangtze Front (CYF) because it takes the form of a slanted S-shape connecting Cheju Island and the Changjiang river mouth^2,13^. The CYF is strongest in the winter when it consists of a northern warm-tongue in the east where high-temperature and high-salinity water of TWW origin expands northwestward and a southern cold-tongue in the west where low-temperature and low-salinity water is transported southeastward by the ECSC^14,15^ (Fig. 1a). The northern warm-tongue is divided into a northern front that develops in the east-west direction at a latitude of 34°N and a southern front that forms in the northwest-southeast direction along isobaths between 50 and 75 m in the Changjiang Bank^14,16^ (Fig. 1a). The CYF frontal circulation pattern is characterized by counter-clockwise (cyclonic) rotating currents along the warm-tongue, namely, westward along the northern front, called the Westward Transversal Current, and southeastward along the southern front, called the Southeastward Outflow^14^. The ECSC, which comes southeast from the east coast of China across the Changjiang Bank, is combined with the southeast frontal jet of the warm-tongue to strengthen the Southeastward Outflow^4^. The warm-tongue weakens as the intrusion of warm water into the Yellow Sea decreases from winter to summer^2^. Several studies on the structure and circulation of the CYF have been conducted because the slanted S-shaped CYF is one of the largest thermohaline fronts in winter associated with the circulation of adjacent water masses in the Yellow Sea and in the East China Sea^2,13^.

The structures and circulations of the CYF have mainly been investigated in relation to the northern front of the northern warm-tongue by using hydrographic and satellite observations and numerical models. In the winter of 2002–2003, the structure of episodic large temperature inversion at the entrance of the Yellow Sea along 34°N was reported to be associated with the northward advection of the CWC water and the westward advection of the Korean Coastal Water^17–19^. Wang *et al*. revealed the physical connection between the westward shift of the warm-tongue and the northern front, wherein the westward shift of the warm-tongue is strengthened when the northern front migrates more southward or grows stronger^20^. Based on *in-situ* hydrographic data, Lie *et al*. disclosed the frontal circulation of the warm-tongue wherein the geostrophic current pattern indicates a cyclonic circulation along the front and the northern front of the warm-tongue shows a wave-like shape during the winter monsoon^14^. Studies have examined the structure of the northern front and associated circulation; however, the southern front of the northern warm-tongue remains largely unexplored despite its significance for heat and material exchanges between the open Pacific and shelf seas.

Thus, in this study, we aim to (1) analyse the structures of the southern front of the warm-tongue and (2) identify relevant frontal circulation from the perspective of heat and material exchanges based on *in-situ* hydrographic observations during two recent cruises, historical (over 50 years) hydrographic observations, and supplementary satellite observations.

## Results

### Circulation and frontal structures observed in February 2017 and April 2018

A cyclonic circulation in relation to the structure of the southern front of the warm-tongue was newly identified from hydrographic observations through two cruises in February 2017 (winter) and April 2018 (spring) (Fig. 1b,c). The currents observed at a depth of 40 m during these cruises after extracting tidal currents are broadly consistent with satellite altimetry-derived surface geostrophic currents averaged over the observation periods (red vs black arrows), yielding known circulation patterns, namely, the TWC and CWC, except for a significant difference in the western entrance of Cheju Strait and the line C05–C10 (Fig. 1b,c). In the western entrance of Cheju Strait, the directly observed mid-depth (40 m) currents follow the CWC path but the surface geostrophic currents do not. Surface geostrophic currents in winter reflect cyclonic circulation in the northern East China Sea; however, surface cyclonic circulation does not exist in the spring owing to the development of thermal stratification. Sea surface heights (SSH) and their differences in areas west of 127°E in spring were generally lower than those in winter; therefore, surface geostrophic currents were weaker in spring than in winter.

**Figure 2:**
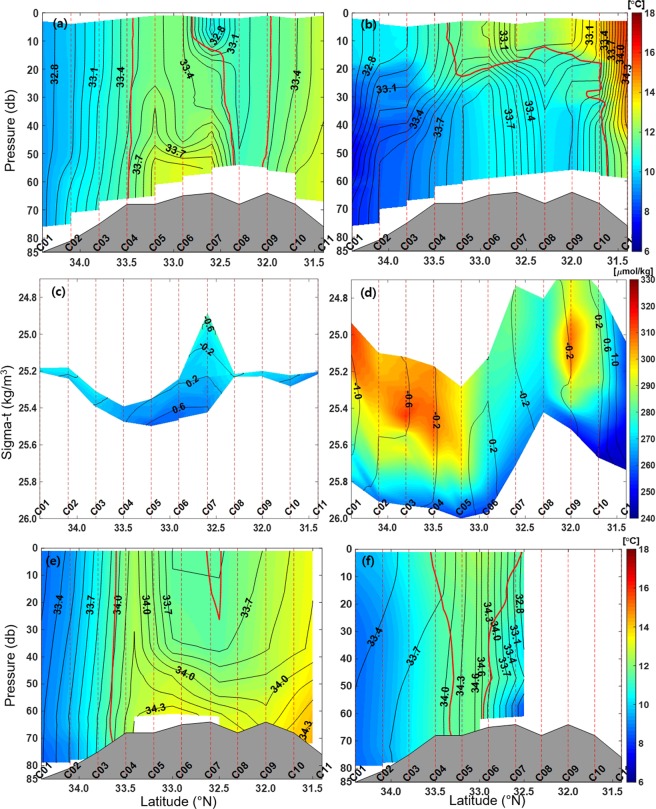
Cross-sectional structures along the C-line of (**a,b**) conservative temperature (Θ, colours) and absolute salinity (S_A_, contours), and (**c,d**) dissolved oxygen (colours) and spiciness (contours) during the (**a,c**) winter (Feb. 2017) and (**b,d)** spring (Apr. 2018) cruises. In (**a,b)**, intervals of contour and label are 0.1 and 0.3 g/kg, respectively. Same as (**a,b**) but for climatological mean in (**e**) Feb. and (**f**) Apr. as derived from historical data (NIFS). The isotherm of 11.5 °C is denoted by thick red solid lines in (**a,b,e,f**). Figures were generated by H.J. Lee using MATLAB R2017b (http://www.mathworks.com).

Alternate intruding frontal structures associated with the cyclonic circulation were observed at lower depths along the cross section of the C-line during both cruises (Fig. 2a–d). In February 2017, relatively high-salinity (>33.4 g/kg) and high-temperature (>11.5 °C) water of TWW origin was found at the lower depths of Stations C04–C07 and in whole water columns of Stations C10–C11. By contrast, low-salinity (<33.4 g/kg) and low-temperature (<11.5 °C) water transported by the ECSC was identified in the water columns of Stations C01–C04 and C08–C09 and upper depths of Station C07. In April 2018, the structure of temperature and salinity is significantly different from that observed in February 2017. Relatively low-temperature (<11.5 °C) water transported by the ECSC occupies whole water columns at Stations C01–C04 and at depths below 20 m at Stations C05–C10. However, relatively high-salinity (>33.4 g/kg) water of TWW origin was still found at the lower depths of Stations C04–C07 and in whole water columns at Stations C10–C11, as observed in February 2017 (Fig. 2a,b). These alternate intruding frontal structures could also be distinguished using DO and π (Fig. 2c,d). The higher the TWW (water originating from the ECSC), the lower (higher) is DO and the higher (lower) is π in both winter and spring. Despite the higher DO of water having a higher proportion of water originating from the ECSC in spring than in winter, alternate intruding frontal structures were consistently found at lower depths during the two seasons. Such cyclonic circulation and frontal structure were also observed from satellite altimetry-derived SSH as described below.

### Seasonal (February vs April) and year-to-year (2017 vs 2018) variations of circulation and frontal structures

**Figure 3:**
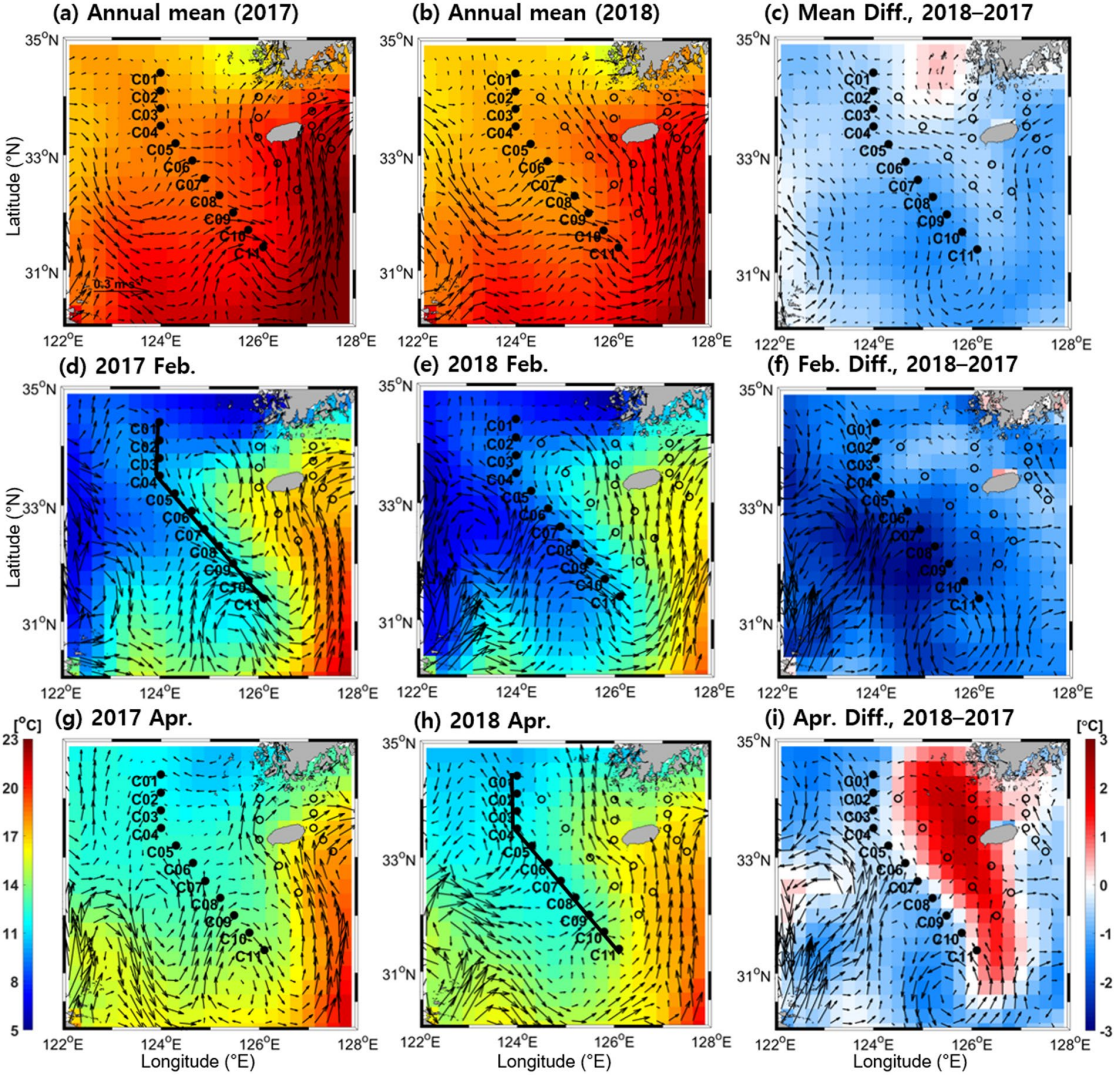
Horizontal distributions of satellite altimeter-derived surface geostrophic current (vector arrows) with sea surface temperature (colours) for annual mean in (**a**) 2017, (**b**) 2018, and (**c**) the difference; Feb. of (**d**) 2017 and (**e**) 2018 and (**f**) the difference; and Apr. of (**g**) 2017 and (**h**) 2018 and (**i**) the difference. Here, all differences are values in 2018 minus those in 2017. Figures were generated by H.J. Lee using MATLAB R2017b (http://www.mathworks.com).

At the surface, the cyclonic circulation found only in February (not April) is consistent with maps of surface geostrophic currents calculated from the satellite altimetry-derived SSH. Surface cyclonic circulation was seen in the maps of surface geostrophic currents in February of both 2017 and 2018 (Fig. 3d,e) but was not clear in April of both 2017 and 2018 (Fig. 3g,h). Annual mean surface currents in both years also do not show cyclonic circulation (Fig. 3a,b), indicative of its intermittency at surface circulation (e.g., winter only). In the annual mean, the TWC pattern is confirmed but the CWC pattern is not (Fig. 3a,b). The northward-flowing TWC extended to the northward flow (CWC) to the west of Cheju Island, which did not continue to flow eastward into the Cheju Strait but instead flowed westward along latitude 34°N (Transversal Current) and southeastward along the southern front (nearly aligned with the C-line) in February (Fig. 3d,e). Year-to-year differences in annual mean, February mean, and April mean surface currents are primarily limited to the western East China Sea and not the northern and eastern East China Sea (Fig. 3c,f,i).

The sea surface temperature (SST) of both years commonly showed well-developed S-shaped surface fontal structures only in February (Fig. 3d,e vs Fig. 3g,h). The surface frontal structures are weakened owing to seasonal warming in April and the annual mean SSTs of both years (Fig. 3a,b,g,h). The SST in 2018 was lower than that in 2017 over the whole East China Sea except for the coastal area north of 34°N in the southwest of the Korean Peninsula (Fig. 3c). The February SST was lower in 2018 by 1–3 °C owing to the southeastward extension of colder water transported by the ECSC compared to that in 2017 (Fig. 3f). The April SST was higher along the TWC and related currents in 2018 by 1–3 °C owing to the northward transport of warmer TWW compared to that in 2017 (Fig. 3i). Detailed water properties along the C-line during the two cruises in February 2017 (winter) and April 2018 (spring) and the consistent frontal structure observed from the historical hydrographic data are described in the following section.

### Water properties and frontal structures from historical 58-year observations (1963–2019)

Frontal structures with alternate intruding patterns of water properties are also observed from the historical February and April observations from the National Institute of Fisheries Science (NIFS) (Figs. 1d, 2e,f). In February, high-temperature (>11.5 °C) and high-salinity (>33.7 g/kg) water of TWW origin was found in the whole water columns at Stations C03–C05 and C09–C11 and at lower depths of Stations C05–C09 (Fig. 2e). In contrast, low-temperature (<11.5 °C) and low-salinity (<33.7 g/kg) water transported by the ECSC was found in the whole water columns of Stations C01–C03. At upper depths of Stations C06–C08, low-salinity (<33.7 g/kg) water is clearly identified, but low-temperature (<11.5 °C) water is only found near Station C07 (Fig. 2e). In April, high-temperature (>11.5 °C) and high-salinity (>33.7 g/kg) water of TWW origin was found in the whole water columns of Stations C04–C06, in contrast with the low-temperature (<11.5 °C) and low-salinity (<33.7 g/kg) water transported by the ECSC found in the whole water columns of Stations C01–C03 and C06–C07 (Fig. 2f). The horizontal structure of temperature (Θ) and salinity (S_A_) observed at 40-m depth in February shows that high-temperature and high-salinity water originating from the TWW intrudes in a direction crossing Stations C05–C07 from east to west, and low-temperature and low-salinity water transported by the ECSC intrudes in a direction crossing Stations C07–C10 from west to east (Fig. 1d).

The cross-sectional structures from the historical February and April observations are remarkably consistent with those observed in February 2017 and April 2018 despite the slightly different position and intensity of the front. In February, the dome-like structures of high-salinity and high-temperature (high-π) water of TWW origin are found at the lower depths of Stations C05–C07 despite the slightly higher contrast in the historically observed properties (Fig. 2e). Although low-salinity and low-temperature (low-π) water transported by the ECSC found at the upper depths of Stations C06–C09 is located farther north in historical observations, the overall frontal structures are consistent between February 2017 and historical observations (Fig. 2e). In April, the alternate intruding patterns of temperature and salinity at lower depths along the C-line are consistently found in both 2018 and historical cruises, although thermal stratification is clearly enhanced during April 2018 probably owing to interannual variations of source water properties (Fig. 2f).

## Discussions and Conclusion

In this study, semi-permanent cyclonic circulation in the lower layer of the southern front of the warm-tongue in the northern East China Sea was observed for the first time from two cruises and historical hydrographic data along with supplementary satellite data during both winter and spring. In February, the cyclonic circulation consisting of the northward flowing CWC, westward flowing Transversal Current, and Southeastward Outflow is clearly seen even from surface geostrophic currents in a consistent form with the alternate intruding frontal structures of hydrographic observations along the C-line (Figs. 2e, and 3d,e). Although surface geostrophic currents are smeared owing to enhanced thermal stratification in April, the alternate intruding frontal structures produced by the cyclonic circulation are still maintained along the C-line (Figs. 2f, and 3g,h).

In the lower layer between Stations C04–C07 along the southern front, high-temperature and high-salinity (high-π) water of TWW origin intrudes in the direction crossing the front from east to west (Figs. 2e,f and 3d,e). On the other hand, low-temperature and low-salinity (low-π) water of ECSC origin penetrates in the direction crossing the front from west to east in the upper layer between Stations C07–C10 during the two seasons. The flows crossing the front between Stations C07–C10 are also consistent with the ECSC (named as the Yellow Sea Coastal Current in Yuan *et al*.) flowing southeastward toward the shelf edge southwest of Cheju Island from winter to spring as revealed by ocean colour and SST data^9^. On the basis of the numerical model results, cross-shelf circulation during winter is believed to be primarily due to the northerly monsoonal winds^8^. The numerical study shows the currents flowing counter-clockwise across the 50 m isobaths from west to east at the shelf edge southwest of Cheju Island in February and April under no wind-stress curl and surface wind forcing conditions^8^. Thus, our observational results support the results of this previous modelling study, although that model did not describe the flow across the 50 m isobaths from east to west in relation to the cyclonic circulation in the lower layer. This cross-frontal circulation brings the materials from the shallow Changjiang Bank into the southwestern part of Cheju island or the Okinawa Trough^8^. Some of the materials return to the Yellow Sea by joining the CWC and YSWC to the southwest of Cheju Island^8^. The flows crossing the front in the lower layer between Stations C04–C07 first reported in this study play an important role in local material (as well as heat) transport and biogeochemistry. This flow crossing the front in the lower layer is connected from northeast to southwest during both February 2017 and April 2018, and it may or may not be relevant to the northwestward flowing YSWC to the west of 124°E that is reported by Lie *et al*. and Yu *et al*.^6,7^. This flow is most likely related to high-density water in the warm-tongue formed via diapycnal mixing between low-temperature water of northern origin and high-salinity water of southern origin.

The difference between the satellite-derived surface geostrophic currents and the currents observed at 40 m is linked to the penetration of high-π water of TWW origin into the lower layer of the C-line to produce different geostrophic structures between surface and bottom layers (Fig. 1b,c). The observed current speeds of southeastward flow along the southern front are 1–30 cm s^−1^ in February 2017 and 2–16 cm s^−1^ in April 2018; these are comparable to those reported from surface drifter observations^16^. Possible forcing mechanisms for semi-permanent cyclonic circulation are discussed. The water transported from the centre of the cyclonic circulation west of Cheju Island outward by the Southeastward Outflow crossing the C-line is high-π water of TWW origin with significantly higher density (sigma-t) than that of TWW source water (e.g., 25.4 vs 25.2 kg m^−3^) (Fig. 2c). Such an increase in density is likely caused by wintertime surface cooling and vertical diapycnal mixing of high-salinity water of TWW origin before isopycnal mixing with low-π water transported by the ECSC^14,16^. West of Cheju Island, the confluence of high- and low-π water forms high-density water (>25.6 kg m^−3^) in patch-shaped zones between 33–34°N and 124–126°E in February, resulting in vertical compression of the water column corresponding to low SSH. Previous *in-situ* hydrographic observations also show the patch-shape of the horizontal density structure, unlike the northwestward intrusion pattern, that is well developed between 33–34°N and 124–126°E in winter^14^. Water movement is adjusted by the lowered SSH-derived cyclonic circulation around the centre geostrophically; this is consistent with satellite-derived surface geostrophic currents seen in February (Fig. 3d,e). In April, although the warm-tongue structure retreats eastward with seasonal surface warming^16^, the cyclonic geostrophic circulation formed by high-salinity and low-temperature water is still maintained at lower depths around the centre. The horizontal distribution (not shown here) of density at 50-m depth also indicates that high-density water (>26.0 kg m^−3^) is located between 33–34°N and 124–126°E in April.

The semi-permanent cyclonic circulation may also be attributable to the conservation of potential vorticity with respect to the change of bottom topography^21^. As the distance of the isobaths between 50 and 75 m narrows along the C-line from Station C04 to Station C09, as indicated by, for example, the steeper slope of 0.00044 (C04) to 0.00076 (C09), bottom relief becomes significant at southern stations such as C09, inducing cyclonic circulation to conserve the potential vorticity conservation owing to water column stretching (increasing relative vorticity). More quantitatively, the changes of the Coriolis parameter (planetary effect) and water depth (stretching of the topographic effect) along the C-line between Stations C06 and C11 are compared. The increase in relative vorticity due to the topographic effect (1.31 × 10^−5^ s^−1^) for fixed latitude is approximately four times that due to the planetary effect (3.24 × 10^−6^ s^−1^) for fixed water depth.

**Figure 4:**
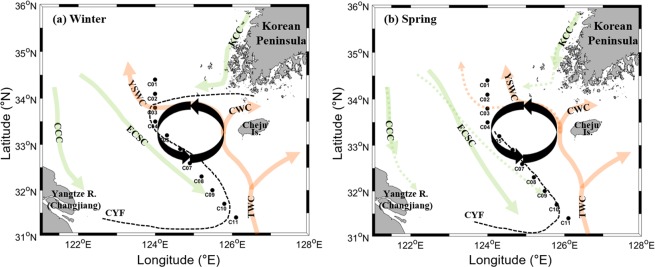
Schematic diagram illustrating cyclonic circulation in the lower layer of the southern front in (**a**) winter and (**b**) spring along with surface currents and fronts. For information on the acronyms, dashed lines, and coloured arrows, see the caption of Fig. 1. Figures were generated by H.J. Lee using MATLAB R2017b (http://www.mathworks.com) and modified using Microsoft PowerPoint (http://www.microsoft.com).

The cyclonic circulation in the lower layer of the southern front is schematized in Fig. 4 along with surface currents and fronts. The cyclonic circulation is maintained in the lower layer west of Cheju Island while surface currents and the CYF shift from winter to spring. The semi-permanent cyclonic circulation found at lower depths in the northern East China Sea presented herein likely results from horizontal frontal water exchange and vertical heat exchange through the sea surface. The high-π water of TWW origin and the distinctively contrasting low-π water transported by the ECSC form a prominent thermohaline front that shapes the alternate intruding frontal structure along the southern front of the warm-tongue. Considering the patch-shaped high-density water formed west of Cheju Island in winter and the potential vorticity conservation, the cyclonic circulation pattern will be maintained consistently even if the YSWC occurs intermittently in winter. These findings will hold for any thermohaline frontal zone in a shallow and broad shelf where waters of contrasting properties (high- vs low-π) having nearly the same density are widely exchanged and seasonal warming/cooling is severe, and they have significant implications for the shelf ecosystem.

## Methods

Vertical profiles of temperature, salinity, density, and dissolved oxygen were observed at 16 and 21 stations in the region using a Conductivity-Temperature-Depth (CTD) sensor (Sea-Bird Scientific, SBE 911plus) and oxygen sensor (Sea-Bird Scientific, SBE 43) from two cruises of R/V *Onnuri* during February 6–14, 2017 (winter), and April 20–30, 2018 (spring), respectively (Fig. 1). We used the CTD and oxygen data collected at stations along the C-line (closed circles in Fig. 1). Vertical profiles of horizontal current were measured with a depth bin size of 4 and 8 m from the ship-mounted 150- and 75-kHz acoustic Doppler current profiler (ADCP) during the winter and spring cruise periods, respectively. To supplement the data acquired from the two cruises, historical 58-year hydrographic data (1963–2019) from the NIFS collected in February at five stations and April at seven stations near the C-line were used (indicated by filled squares in Fig. 1b,c). The historical data for 15 standard depths (surface, 10, 20, 30, 50, 75, 100, 125, 150, 200, 250, 300, 350, 400, and 500 m) of the unevenly distributed stations are archived, where 4–49 and 1–42 vertical profiles of temperature and salinity at each station are available for February and April (mostly north of 32.5°N), respectively. In addition to the *in-situ* observations, daily and monthly mean gridded data with 0.25° horizontal resolution of satellite altimetry-derived SSH and satellite microwave-based SST from the Copernicus Marine Environment Monitoring Service (CMEMS) were used (http://marine.copernicus.eu/services-portfolio/access-to-products). The Finite Element Solution 2014 (FES2014) tide model was used for the tidal correction of SSH data in CMEMS^22^. Recently, the uncertainty of satellite altimetry data was greatly reduced to a degree of only a few centimetres in shallow shelves, particularly the study area^23,24^. Considering the horizontal resolving scale of the altimetry data (few tens of kilometres) and the uncertainty of the satellite-derived SSH (only a few centimetres), the uncertainty of satellite-derived geostrophic current is of the order of 0.1 cm s^−1^. This means that geostrophic currents with magnitude of 1–10 cm s^−1^ are well beyond the error range. Because the ageostrophic components in the satellite-derived SSH can be mostly removed by the correcting procedure of using the FES2014 model and eliminating global high-frequency barotropic motion, the corrected SSH products would well support the geostrophic balance^22^. Bathymetry data with 15 arc-second intervals from GEBCO Compilation Group (2019) GEBCO 2019 Grid (10.5285/836f016a-33be-6ddc-e053-6c86abc0788e) were used in this study.

The temperature, salinity, density, dissolved oxygen, and current data were processed by standard quality control and assurance procedures^25,26^. To control the data quality, CTD data were processed by removing abnormal values (wild editing), correcting the misalignment of the temperature and the conductivity sensors (align CTD), and correcting the thermal inertia effect that occurs when the CTD passes by a thermocline layer (Cell TM)^26,27^. Dissolved oxygen (DO) values were corrected by a regression analysis between sensor values and titration results obtained at six stations in February 2017 and at seven stations in April 2018. The magnetic deviation of −7.2° caused by the compass in the ADCP data collected in the region was corrected. Spikes and density inversion layers were removed from data acquired from both cruises and the NIFS data. The temperature and salinity of all hydrographic data are expressed in units of conservative temperature (Θ) and absolute salinity (S_A_, g/kg) calculated from the *in situ* temperature (ITS-90) and practical salinity (PSS-78), respectively, through the Thermodynamic Equation of SeaWater 2010 (TEOS-10)^28^. Major tidal constituents (M2, S2, K1, O1, N2, K2, P1, and Q1) were removed from the observed currents to extract nontidal currents using a global barotropic tidal model (TPXO8.v1) with horizontal resolution of 1/30° ^29^. Zonal and meridional components of tidal current obtained at a given time and location using the Tidal Model Driver (TMD) program were used to eliminate the tidal currents from the ADCP measurements after validating with independent current measurements in the coastal area. The M_2_ (period of 12.42 h) tidal currents modelled by TPXO8.v1 yield an error of 3.7 cm s^−1^ based on a comparison with those extracted from the depth-averaged ADCP measurements at two locations that were 37 km distant from each other in the same shelf area. The data processing procedure to remove and validate tidal currents using the TPXO8.v1 model are illustrated in detail in Supplementary Figs. S1–S3. The phase difference between the tidal height observed at the nearby tide-gauge station and that modelled by the TPXO8.v1 is 14 min, which is negligible considering the distance of the tide-gauge station to the model grid (~45 km) and the grid size of 3.7 km. The temperature and salinity observed at standard depths of five or seven NIFS stations near the C-line were climatologically averaged for each month over the 58-year period and vertically interpolated at 1-m intervals. The spiciness, π, was used to represent water properties along isopycnal surfaces^11,30^. Surface geostrophic currents were calculated from the satellite altimetry-based SSH data.

## Supplementary information

Supplementary information.
