# Supplementary material for: Observations of the warm-tongue circulation in the northern East China Sea
Source: Sci Rep. 2020 Jan 14;10:276. doi: 10.1038/s41598-019-57148-6 (PMC6959225; doi:10.1038/s41598-019-57148-6)
Supplement: Supplementary file 1 — Supplementary information. [file 41598_2019_57148_MOESM1_ESM.docx]

**Supplementary Information**

**Observations of the warm-tongue circulation in the northern East China Sea**

**Hojun Lee^a^, Kyungjae Lee^a^, SungHyun Nam^a, b*^, and Jae-Hak Lee^c^**

^a^School of Earth and Environmental Sciences, Seoul National University, Seoul, Republic of Korea

^b^Research Institute of Oceanography, Seoul National University, Seoul, Republic of Korea

^c^Korea Institute of Ocean Science and Technology, Busan, Republic of Korea

^*^**Corresponding author**: 1 Gwanak-ro, Gwanak-gu, Seoul 08826, Republic of Korea

E-mail address: namsh@snu.ac.kr (S. H. Nam)

**Table of Contents of the Supplementary Information**

1. Depth-averaged currents observed over semidiurnal (M_2_) tidal cycle in February 2017
2. Barotropic tidal currents modelled over the same period in February 2017
3. Comparison between the latitudinal structures of observed currents averaged over the tidal cycle and model-based de-tided currents


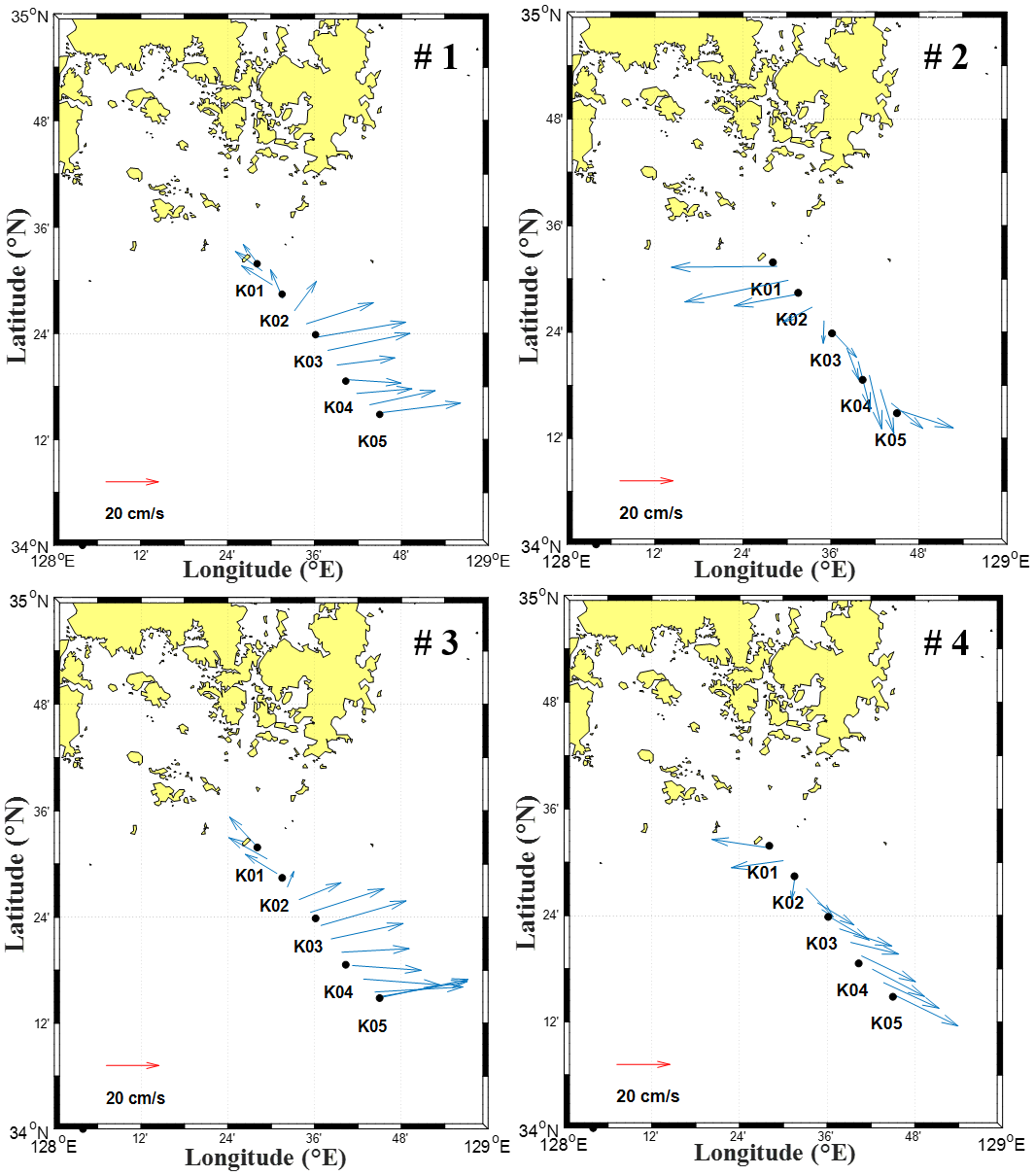


**Figure S1. Depth-averaged currents observed over 13 depth bins from 22.5 to 82.5 m (water depth shallower than 120 m) using Shipboard Acoustic Doppler Current Profilers.** Two round-trip (four cross-sectional) observations were conducted across Stations K01-K05 over two semidiurnal (M_2_, period of 12.42 h) tidal cycles off the southeast coast of Korea from February 14 to 15, 2017 (#1: from 14:07 to 17:07 February 14, #2: from 20:22 to 23:07 February 14, #3: from 02:37 to 05:52 February 15, and #4: from 08:52 to 11:37 February 15). Figures were created using MATLAB R2017b (<http://www.mathworks.com>).


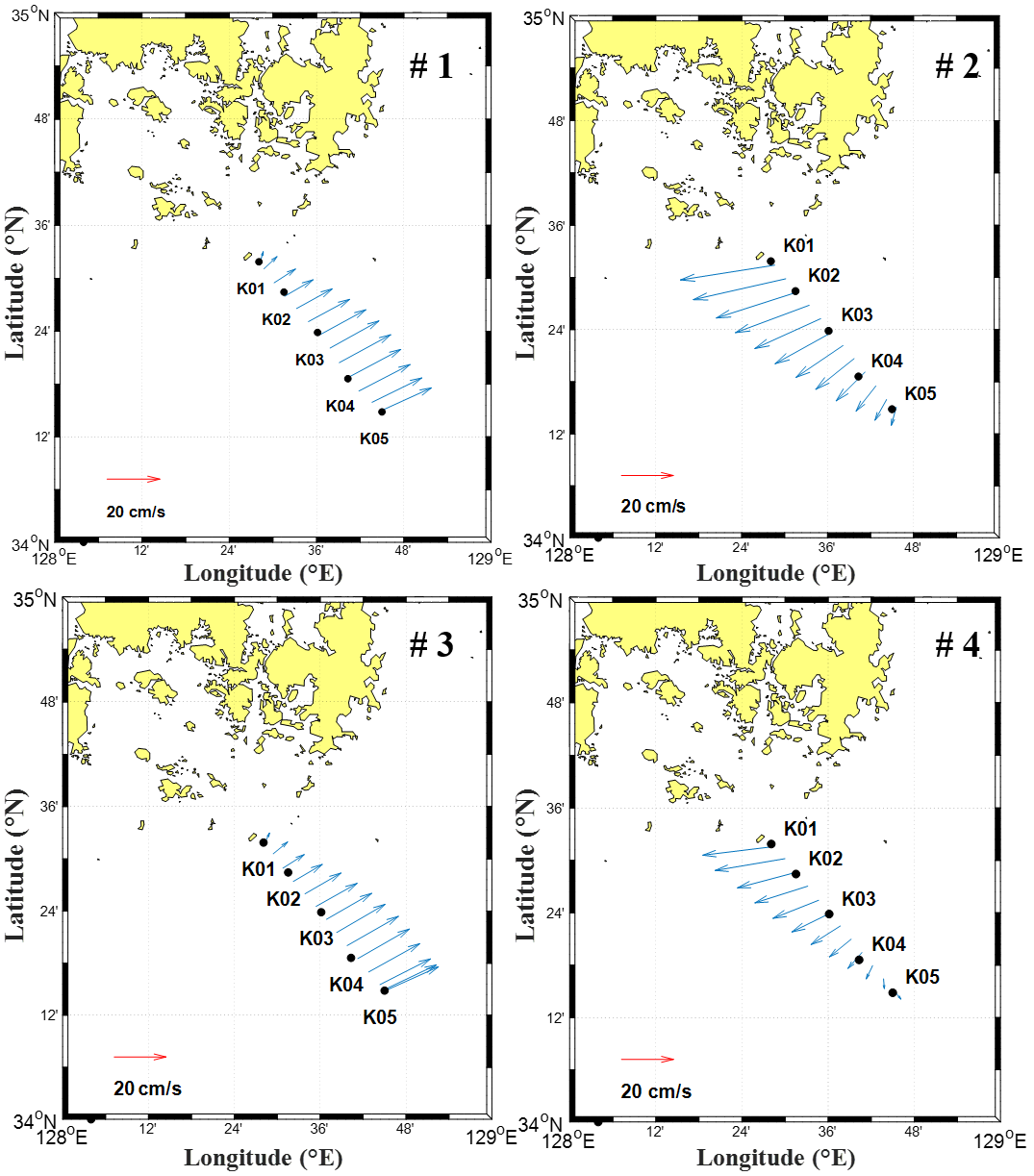


**Figure S2. Barotropic tidal currents obtained by the global barotropic tidal model (TPXO8v.1) at the times and locations corresponding to the observations in February 2017 (Fig. S1).** The tidal currents are the sum of the major tidal constituents (M_2_, S_2_, K_1_, O_1_, N_2_, K_2_, P_1_, and Q_1_) at corresponding dates and times of the observations from #1 to #4 (Fig. S1). Figures were generated using MATLAB R2017b (<http://www.mathworks.com>).


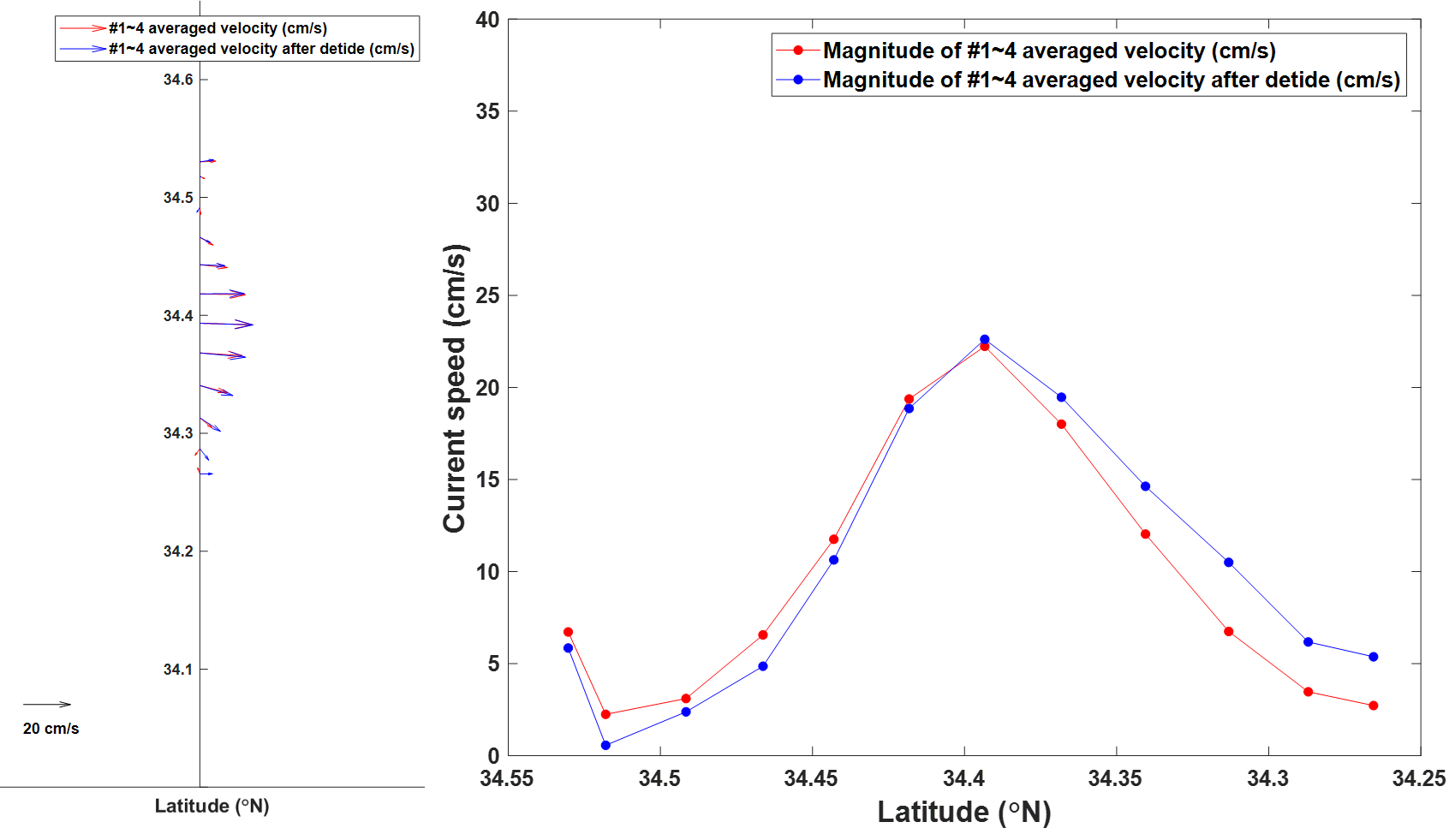


**Figure S3. Comparison between the latitudinal structures of observed currents averaged over the tidal cycle and model-based de-tided currents.** Vector (left) and speed (right) plots showing the latitudinal structure of observed currents averaged over the four cross-sections and the two semidiurnal tidal cycles with (blue) and without (red) the de-tiding process performed using the TPXO8.v1. The typical error of de-tided currents was estimated to 3.7 cm s^-1^ from the mean difference between these two currents. Figures were obtained using MATLAB R2017b (<http://www.mathworks.com>).
